# Mortality Among Patients Undergoing Blood Transfusion in Relation to Donor Sex and Parity: A Natural Experiment

**DOI:** 10.1001/jamainternmed.2022.2115

**Published:** 2022-06-13

**Authors:** Jingcheng Zhao, Arvid Sjölander, Gustaf Edgren

**Affiliations:** 1 Clinical Epidemiology Division, Department of Medicine, Solna, Karolinska Institutet, Stockholm, Sweden; 2 Department of Medical Epidemiology and Biostatistics, Karolinska Institutet, Stockholm, Sweden; 3 Department of Cardiology, Södersjukhuset, Stockholm, Sweden

## Abstract

**Importance:**

Prior evidence suggests that plasma and platelet transfusions from female and parous donors are associated with adverse clinical outcomes, which has led to the predominant use of male donors for plasma and platelets in many countries. However, studies on red blood cell transfusions have been conflicting.

**Objective:**

To determine whether blood donor sex and parity affect mortality of patients undergoing transfusion with red blood cells.

**Design, Setting, and Participants:**

This cohort study used nationwide blood bank and health register data in Sweden and involved a natural experiment created by donor sex and parity being concealed and randomly allocated. Patients were included if they were 18 to 90 years old, did not have a history of blood transfusion, and received a transfusion between January 1, 2010, and December 31, 2017. Patients were followed up from their first red blood cell transfusion until death, emigration, or end of study (June 30, 2018). Data analysis was performed between June 15 and December 15, 2021.

**Exposures:**

(1) Female vs male donors and (2) parous or nonparous female vs male donors.

**Main Outcomes and Measures:**

Overall survival up to 2 years estimated using inverse probability–weighted Kaplan-Meier estimates and relative risk for additional transfusions within 24 hours.

**Results:**

Among the 368 778 included patients (mean [SD] age, 66.3 [17.7] years; 57.3% female), 2-year survival differences comparing red blood cell transfusions from female and parous donors to male donors were −0.1% (95% CI, −1.3% to 1.1%) and 0.3% (95% CI, −0.6% to 1.2%), respectively. No statistically significant survival differences were observed regardless of patient sex or age. Median (IQR) hemoglobin counts for female donors (13.5 [13.0-14.0] g/dL) were lower than for male donors (14.9 [14.4-15.5] g/dL). Red blood cell transfusions from female donors were associated with a relative risk of 1.12 (95% CI, 1.08-1.17) for additional transfusions within 24 hours but not after adjusting for donor hemoglobin counts (relative risk, 1.03; 95% CI, 0.98-1.08). Pretransfusion patient characteristics were naturally distributed as-if randomized.

**Conclusions and Relevance:**

In this nationwide cohort study involving a natural experiment, after accounting for the lower hemoglobin values in blood from female donors, patients undergoing transfusion with blood from female or parous donors did not have higher 2-year mortality compared with recipients of blood from male donors.

## Key Points

**Question:**

Does blood donor sex or parity affect mortality of patients undergoing transfusion?

**Findings:**

In this nationwide cohort study involving a natural experiment of 368 778 patients undergoing red blood cell transfusion, the 2-year survival differences comparing transfusions from female and parous donors with male donors were both not statistically significant at −0.1% and 0.3%, respectively. Receiving blood from female donors was associated with an increased risk of additional transfusions owing to female donors having lower hemoglobin counts.

**Meaning:**

Patients undergoing transfusion with blood from female or parous donors did not have a higher mortality compared with patients undergoing transfusion with blood from male donors; however, female blood units tended to have lower hemoglobin content.

## Teaser

This nationwide cohort study involving a natural experiment assesses whether blood donor sex and parity affect mortality of patients undergoing transfusion with red blood cells.

## Introduction

Female donor sex and parity are established risk factors for transfusion-related acute lung injury (TRALI) following plasma and platelet transfusions, which is a leading cause of transfusion-related mortality.^1,2,3,4^ Preferential use of male plasma and platelets in many countries has reduced the incidence for TRALI.^1,5^ However, recent observational studies have also shown increased mortality in patients receiving red blood cell transfusions from female donors,^6,7^ in male patients receiving transfusions of red blood cells from previously pregnant but not nonparous female donors,^8^ and in sex-mismatched transfusions.^7,9,10,11^ These findings are conflicting with other studies of red blood cell transfusions, which reported no such associations.^12,13,14^ However, it is not fully understood why results from previous studies have been conflicting, both between and within studies, despite being conducted in similar settings with similar methods.^6,7,9,10,11,12,13,14^ Limiting the use of red blood cell units from female donors may seriously jeopardize the blood supply.

Previous studies have not accounted for comparatively lower hemoglobin counts in female donors compared with male donors. Head-to-head comparisons are therefore likely inappropriate because units of blood from female donors essentially constitute lower “doses” of hemoglobin. The difference in hemoglobin content may create a type of time-dependent confounding known as treatment-confounder feedback, which can lead to unpredictable bias when using standard regression models.^15^ This is a source of bias that may explain the conflicting results both between and within previous studies.^15,16,17,18,19^

In this nationwide cohort study involving a natural experiment, we assessed if lower hemoglobin counts in female donors may result in treatment-confounder feedback and be a previously unrecognized source of bias. Furthermore, we assessed if blood donor parity and sex were naturally assigned as-if randomized and constituted a natural experiment, as blood allocation was blinded to donor sex or parity. Based on this, we emulated a nationwide randomized clinical trial to study the effect of donor sex and parity on mortality of patients undergoing transfusion of red blood cells, using appropriate analytical methods that mitigate bias owing to treatment-confounder feedback.

## Methods

### Study Design

In a nationwide study of transfusion-naive persons receiving red blood cell transfusions from January 1, 2010, through June 30, 2018, we separately assessed overall survival in 2 sets of red blood cell transfusion strategies: (1) female vs male donors and (2) nonparous or parous female vs male donors. No other outcomes or blood products were assessed.

To minimize confounding, we exploited a natural experiment in the blood allocation process with respect to donor sex and parity.^20^ The rationale for the natural randomization is that the choice of red blood cell units was solely based on blood group and otherwise followed a first-in, first-out policy. Donor sex and parity were also neither labeled on the blood product nor routinely communicated to the treating physician or patient. Therefore, donor sex and parity are expected to be as-if randomized with respect to important patient characteristics and are effectively double blinded. However, because both donor and patient characteristics may vary slightly with blood group, geographical region, and calendar year, these 3 factors also need to be accounted for. In analogy with a multicenter study, we collectively refer to blood group, region, and calendar year as center characteristics. After empirically confirming that the distribution of donor sex and parity were de facto independent of patient characteristics and that the balance between treatment arms further improved after adjusting for center characteristics, we used inverse probability weighting to emulate a randomized target trial.^21,22^ In the Supplement, see eMethods 1 for a graphical summary of the method, eMethods 2 for formal definitions, and eTable 1 for the study protocol of the target and emulated trial.

The conduct of this study and waiver for informed consent owing to use of deidentified data only was approved by the Regional Ethics Committee in Stockholm and the Swedish Ethics Review Authority. The Strengthening the Reporting of Observational Studies in Epidemiology (STROBE) reporting guidelines were used.

### Data Sources and Study Population

The study population was defined using data collected from all blood banks and hospitals in Sweden and included all persons who received a first red blood cell transfusion at 18 to 90 years old between 2010 and 2017. Patients were followed up from the time of the first transfusion for a maximum of 2 years or until midnight on the date of death, emigration, end of study (June 30, 2018), or hour of the first transfusion of a unit from an unidentified donor or autologous unit, whichever came first. The TRALI-mitigation policies, such as preferential male plasma use or TRALI-related antibody screening, did not apply to red blood cell donors during the study period. We did not differentiate red blood cell units based on whether they were leukoreduced or irradiated. Universal leukoreduction was gradually introduced in Sweden starting in the mid-1990s.^23^

All blood donors, predonation hemoglobin counts, links between donors and patients, ABO and RhD blood groups, and the date and time of transfusions were extracted from blood bank databases. Unique personal identifiers for both donors and patients undergoing transfusion were used to ascertain comorbidities from the National Patient Register and Cancer Register. Data on migration, sex, and vital status were retrieved from national population registers.^24^ Donor parity was assessed through the Medical Birth Register, containing all births in Sweden with nationwide coverage starting 1973, and the Multi-generation Register starting 1932. Laboratory data were retrieved from all hospitals in Stockholm, Sweden, for all patients treated in this region. Reporting to all data sources used in this study is mandated by Swedish law, ensuring high completeness.

All data were assembled into a previously reported research database denoted the SCANDAT3-S database, which contains data on all electronically recorded donations and transfusions in Sweden from the late 1960s until July 1, 2018, with full nationwide coverage starting in the mid-1990s.^25^ Full coverage of relevant blood tests ordered for all patients in Stockholm (the Swedish capital and largest city), both inpatient and outpatient, was available during the entire follow-up. Laboratory data and diagnosis data were only used for evaluating the assumption of natural randomization.

### Assessment of Natural Randomization

As a measure of whether blood donor sex and parity were naturally distributed as-if randomized, we assessed pretransfusion patient characteristics across treatment arms. We used descriptive statistics both at baseline and throughout follow-up for patient demographics; comorbidities, including cancer and 31 comorbidities as defined in the Elixhauser Comorbidity Index; results of the most recent laboratory tests (≤1 week before transfusion) such as complete blood cell count, C-reactive protein, and serum creatinine; and initial transfusion indication. The Elixhauser Comorbidity Index and transfusion indication was derived from discharge diagnoses using the eighth, ninth, and tenth revisions of the *International Classification of Diseases*.^26,27,28^ For inpatient episodes, the date of admission was used as date of diagnosis. To assess the influence of center characteristics, we compared absolute standardized mean differences of baseline patient characteristics across treatment arms, before and after weighting with the inverse probability of donor sex or parity accounting for center characteristics. This was calculated as the absolute value of the difference in means, divided by the variance of the compared groups.^29^ Weighting adjusts for the center characteristics by balancing them across treatment arms.^15,30^ Full details and additional diagnostics are available in eMethods 2 in the Supplement.

### Treatment-Confounder Feedback Owing to Donor Hemoglobin Counts

Treatment-confounder feedback is the phenomenon by which a confounder is affected by previous treatment. Herein, treatment-confounder feedback between female donor sex (the treatment) and the number of transfusions (the confounder) is expected, because female red blood cell units tend to contain less hemoglobin, which may result in a need for additional transfusions. The number of transfusions is a critical confounder because it is directly related to being exposed and is an important predictor for comorbidity and death. Therefore, the number of transfusions is both a mediator for the relation between previous transfusion and death, but also a confounder for the present transfusion and death. This cannot be adjusted for using standard regression models such as Cox regression. While standard regression models would correctly remove confounding for the present transfusion and death, it would also attenuate any effect that prior transfusions may have on death, which creates bias toward the null. Additionally, such an adjustment creates a spurious (noncausal) statistical association between the previous transfusion and death through unmeasured confounders of the number of transfusions and death, which can produce a bias away from the null.^15^ These unmeasured confounders include frailty and comorbidity, which are common causes of both needing a transfusion and death. Bias owing to treatment-confounder feedback is therefore unpredictable and can result in a net bias effect both toward and away from the null.^15^

To directly demonstrate the presence of treatment-confounder feedback, we calculated the relative risk of receiving additional red blood cell units in the first 24 hours for patients receiving female-donor units compared with male-donor units only, with and without adjusting for donor hemoglobin count. To accommodate the high prevalence of double-unit transfusions during the study period,^28^ donor sex was assessed for a subcohort (62%), who received 2 transfusions within 6 hours, and additional transfusions were assessed in the ensuing 18 hours after the second transfusion. See eMethods 2 in the Supplement for additional details and a formal discussion of treatment-confounder feedback.

### Statistical Analyses

We emulated a target trial by exploiting the natural randomization of donor sex and parity. To mitigate bias owing to treatment-confounder feedback, we used cloning, censoring, and inverse probability weighting. This approach has been used extensively within pharmacoepidemiology and is conceptually identical to a marginal structural model.^15,22,31,32,33,34^ For a nontechnical summary, as well as a detailed, formal discussion of the method and a directed acyclic graph, see eFigure 1 in the Supplement.

In brief, each patient was cloned and assigned every treatment strategy and subsequently censored when they no longer adhered to the treatment strategy. To account for selection bias owing to censoring of nonadherent patients, patients who continued to adhere to the treatment strategy were time-varyingly weighted upward. At every transfusion, patients who received the transfusion of the assigned donor characteristic with the probability *p* would henceforth be upweighted by a factor of 1/*p*. These probabilities were estimated nonparametrically from administrative database data on the calendar year, region, and the patient blood group. Because a transfusion could change the need for a future transfusion already by the next hour, follow-up was divided into hourly periods. Finally, the treatment strategies were compared using Kaplan-Meier curves estimated from the weighted populations. Under the verifiable assumption that the allocation of blood units is random after adjusting for center characteristics, the estimated survival difference is an unbiased estimate of the causal effect that would be observed in a randomized trial with full compliance.

To account for it being less likely to receive many consecutive units from donors with a less common characteristic, adherence was assessed up to the number of units that corresponded to the 99th percentile of the least common arm and to the 99.9th percentile in sensitivity analyses. Corresponding 95% CIs were estimated using bootstrapping with 1000 runs.^35^ We did not separately account for censoring owing to autologous red blood cell transfusions or units from nonidentified donors because it was rare (n = 564 [0.15%]). All data processing and statistical analyses were performed with SAS, version 9.4 (SAS Institute).

## Results

### Cohort Construction

**Figure 1.**
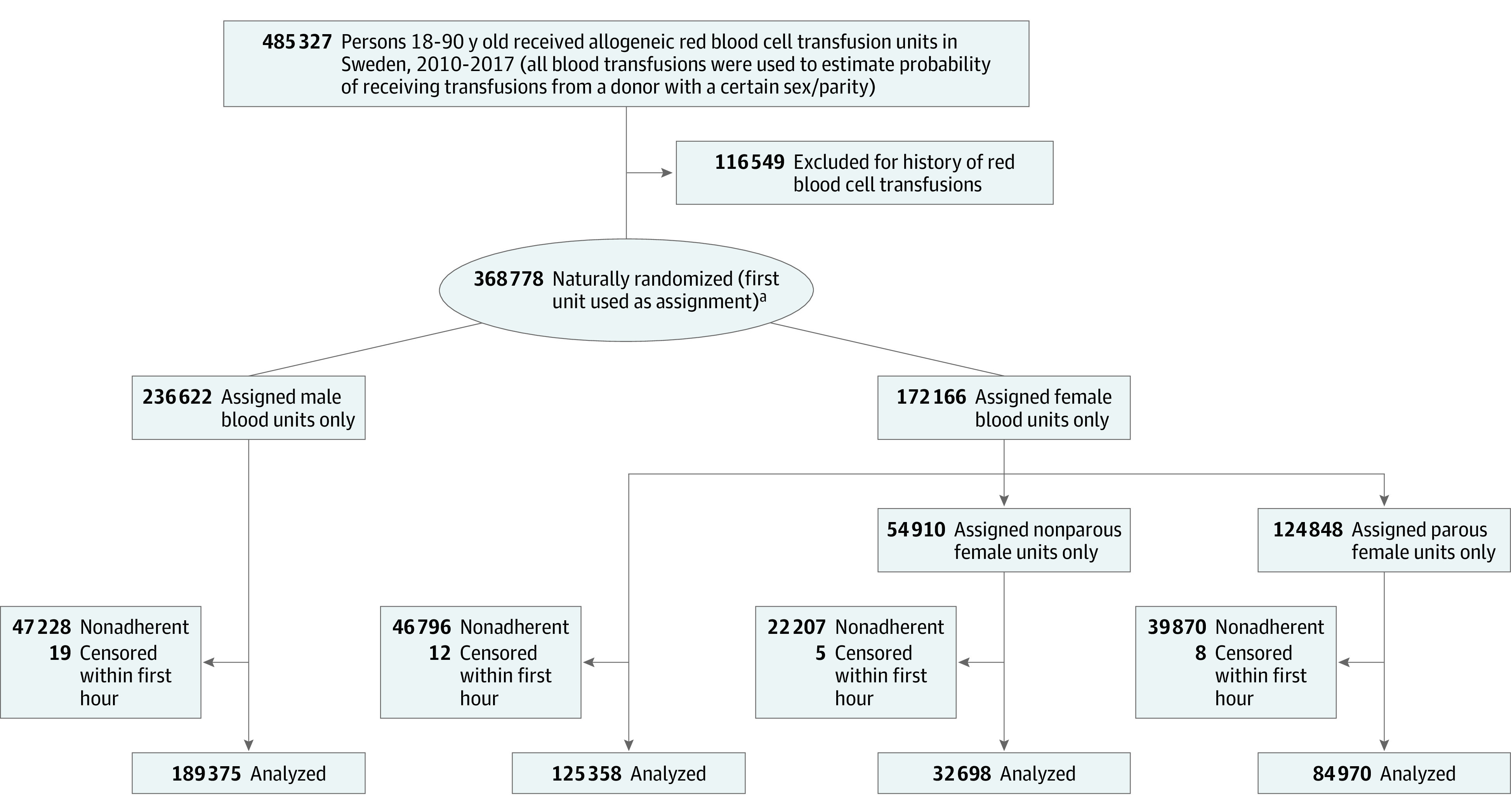
Cohort Flowchart ^a^Used to assess natural randomization.

From a total of 485 327 red blood cell recipients, 368 778 patients without a history of red blood cell transfusions were identified. In total, 1 936 757 red blood cell units from 402 190 donors were transfused during follow-up. Among the patients available for analysis, 189 375 received a red blood cell unit from a male donor as their first unit, and 125 358 received a female-donor unit. Of the latter, 32 698 received a unit from a nonparous female donor, and 84 970 received a unit from a parous female donor. The cohort construction is shown in Figure 1. Inverse probability–weighted analyses were conducted per hour of follow-up, totaling 1 852 976 482 person-hours for donor sex and 1 894 360 834 person-hours for donor parity. Descriptive statistics for transfusions and events are available in eTable 2 in the Supplement.

### Assessment of Natural Randomization

Age, sex, proportion born outside of Sweden, year of first transfusion, blood group, transfusion indication, prevalence of comorbidities, and pretransfusion blood chemistry were similar across treatment arms, both at baseline and throughout follow-up (Tables 1 and 2). Absolute standardized mean differences of extended baseline covariates before and after weighting for center characteristics are presented in eFigures 2 and 3 in the Supplement. Of all 31 Elixhauser Comorbidity Index groups across all treatment arms, the absolute standardized difference compared with male donor strategy was at most 2.8% and decreased to 1.2% after weighting (additional diagnostics are available in eFigures 4 and 5 in the Supplement).

**Table 1.** Pretransfusion Cohort Characteristics at Baseline^a^

Patient characteristic	No. (%)			
	Male blood only (n = 236 622)	Female blood only		
		All (n = 172 166)	Nonparous (n = 54 910)	Parous (n = 124 848)
Age, mean (SD), y	66.0 (17.9)	65.9 (17.8)	65.6 (17.7)	65.9 (17.9)
Sex				
Female	135 749 (57)	98 427 (57)	31 139 (57)	71 514 (57)
Male	100 873 (43)	73 739 (43)	23 771 (43)	53 334 (43)
Born outside of Sweden	36 077 (15)	26 780 (16)	9151 (17)	19 077 (15)
Follow-up in years, median (IQR)	2 (1-2)	2 (1-2)	2 (1-2)	2 (1-2)
Year of first transfusion (IQR)	2013 (2011-2015)	2013 (2011-2015)	2013 (2011-2015)	2013 (2011-2015)
Initial transfusion indication				
Trauma	39 465 (17)	28 520 (17)	8851 (16)	20 652 (17)
Obstetric	18 234 (8)	13 307 (8)	4245 (8)	9867 (8)
Gastrointestinal hemorrhage	21 752 (9)	15 867 (9)	5779 (11)	11 180 (9)
Vascular and/or thoracic surgery	20 320 (9)	14 753 (9)	4583 (8)	10 807 (9)
Cancer surgery	19 956 (8)	14 703 (9)	4858 (9)	10 543 (8)
Other surgery	31 803 (13)	22 734 (13)	7106 (13)	16 480 (13)
Hematological disease	18 189 (8)	13 236 (8)	4111 (7)	9636 (8)
Other malignant disease	27 894 (12)	20 577 (12)	6651 (12)	14 834 (12)
Others or unknown	39 009 (16)	28 469 (17)	8726 (16)	20 849 (17)
Comorbidities				
Any cancer	88 148 (37)	64 858 (38)	21 055 (38)	46 664 (37)
Deficiency anemia	18 996 (8)	13 630 (8)	4192 (8)	9932 (8)
Kidney failure	11 279 (5)	8096 (5)	2543 (5)	5840 (5)
Liver disease	9265 (4)	6795 (4)	2273 (4)	4908 (4)
Elixhauser Comorbidity Index score, median (IQR)	2 (1-4)	2 (1-4)	2 (1-4)	2 (1-4)
Blood chemistry, mean (SD)^b^				
Hemoglobin, g/L	8.9 (1.9)	9.0 (1.9)	9.0 (1.9)	9.0 (1.9)
Leukocytes, per mm^3^	10 500 (14 000)	10 300 (12 270)	10 160 (10 860)	10 390 (13 170)
Platelets, per mm^3^	259 820 (138 210)	259 340 (137 340)	259 030 (137 170)	258 900 (137 840)
Serum creatinine, mg/dL	1.1 (1.0)	1.1 (1.0)	1.1 (1.0)	1.1 (1.0)
C-reactive protein, mg/L	67 (90)	66 (89)	66 (89)	66 (89)
Blood group				
A+	93 438 (39)	64 515 (37)	21 077 (38)	46 233 (37)
A–	16 602 (7)	12 540 (7)	3645 (7)	9392 (8)
B+	21 973 (9)	16 552 (10)	5475 (10)	11 854 (9)
B–	3589 (2)	2983 (1)	920 (0)	2188 (1)
AB+	9723 (4)	7430 (4)	2362 (4)	5423 (4)
AB–	1767 (1)	1419 (1)	428 (1)	1048 (1)
O+	76 426 (32)	55 732 (32)	17 984 (33)	40 272 (32)
O–	13 104 (6)	10 995 (6)	3019 (5)	8438 (7)

^a^ All randomized patients are included, and sums do not add up to totals because more than 1 exposure could be assigned at baseline (see Figure 1 for details on patient selection). eFigures 2 and 3 in the Supplement compare with and without adjustment for center characteristics, including all 31 Elixhauser Comorbidity Index categories.

^b^ Blood chemistry was available in Stockholm, Sweden, only (n = 68 763 [17%]).

**Table 2.** Patient Characteristics Before Each Transfusion During Follow-up^a^

Patient characteristic	No. (%)			
	Transfusions from male donors (n = 1 135 322)	Transfusions from female donors		
		All (n = 801 435)	Nonparous (n = 240 155)	Parous (n = 561 261)
Age, mean (SD), y	66.5 (16.4)	66.4 (16.4)	66.1 (16.4)	66.6 (16.4)
Sex				
Female	548 301 (48)	385 998 (48)	114 151 (48)	271 837 (48)
Male	587 021 (52)	415 437 (52)	126 004 (52)	289 424 (52)
Born outside of Sweden	163 210 (14)	118 357 (15)	37 500 (16)	80 856 (14)
Year of transfusion (IQR)	2013 (2011-2015)	2013 (2012-2015)	2013 (2012-2015)	2014 (2012-2016)
Transfusion indication				
Trauma	150 889 (13)	107 591 (13)	31 961 (13)	75 626 (13)
Obstetric	46 038 (4)	32 170 (4)	9304 (4)	22 866 (4)
Gastrointestinal hemorrhage	121 344 (11)	85 161 (11)	28 514 (12)	56 644 (10)
Vascular and/or thoracic surgery	113 855 (10)	80 451 (10)	23 680 (10)	56 769 (10)
Cancer surgery	88 871 (8)	63 061 (8)	19 541 (8)	43 518 (8)
Other surgery	107 395 (9)	75 798 (9)	22 487 (9)	53 306 (9)
Hematological disease	129 643 (11)	91 089 (11)	27 006 (11)	64 083 (11)
Other malignant disease	167 678 (15)	118 327 (15)	35 499 (15)	82 826 (15)
Others or unknown	209 609 (18)	147 787 (18)	42 163 (18)	105 623 (19)
Comorbidities^b^				
Any cancer	540 108 (48)	381 575 (48)	115 369 (48)	266 200 (47)
Deficiency anemia	102 868 (9)	72 289 (9)	20 740 (9)	51 548 (9)
Kidney failure	73 141 (6)	51 805 (6)	15 503 (6)	36 299 (6)
Liver disease	67 201 (6)	47 806 (6)	15 087 (6)	32 717 (6)
Elixhauser Comorbidity Index score, median (IQR)	3 (1-4)	3 (1-4)	3 (2-4)	3 (1-4)
Blood chemistry, mean (SD)^c^				
Hemoglobin, g/L	8.8 (1.6)	8.8 (1.6)	8.8 (1.7)	8.8 (1.6)
Leukocytes, per mm^3^	10 290 (14 500)	10 230 (14 290)	10 150 (13 440)	10 280 (14 760)
Platelets, per mm^3^	221 070 (153 000)	220 060 (152 360)	220 690 (152 270)	219 690 (152 410)
Serum creatinine, mg/dL	1.2 (1.0)	1.2 (1.0)	1.2 (1.0)	1.2 (1.0)
C-reactive protein, mg/L	80 (94)	80 (94)	81 (94)	80 (94)
Blood group				
A+	449 502 (40)	300 971 (38)	92 666 (39)	208 304 (37)
A–	80 385 (7)	59 662 (7)	16 516 (7)	43 146 (8)
B+	103 261 (9)	75 728 (9)	23 484 (10)	52 244 (9)
B–	17 321 (2)	13 618 (1)	3899 (0)	9719 (1)
AB+	46 576 (4)	34 560 (4)	10 209 (4)	24 351 (4)
AB–	8277 (1)	6484 (1)	1793 (1)	4691 (1)
O+	365 250 (32)	258 007 (32)	77 997 (32)	180 003 (32)
O–	64 750 (6)	52 405 (7)	13 591 (6)	38 803 (7)

^a^ All red blood cell transfusions for the cohort in Table 1 are included.

^b^ Data on comorbidities were available until December 31, 2017. Missing values were generated for transfusions between January 1 and June 30, 2018 (n = 30 431 [2%]).

^c^ Blood chemistry was available in Stockholm, Sweden, only (n = 339 704 [18%]).

### Assessment of Treatment-Confounder Feedback Owing to Donor Hemoglobin Counts

The median (IQR) hemoglobin count was higher among male donors (14.9 [14.4-15.5] g/dL) than female donors (13.5 [13.0-14.0] g/dL). Receiving red blood cell units from female donors was associated with a 12% increased risk of an additional red blood cell transfusion within 24 hours compared with male donors (relative risk, 1.12; 95% CI, 1.08-1.17). However, the difference was no longer statistically significant after adjusting for donor hemoglobin counts (relative risk, 1.03; 95% CI, 0.98-1.08).

### Female vs Male Donors

**Figure 2.**
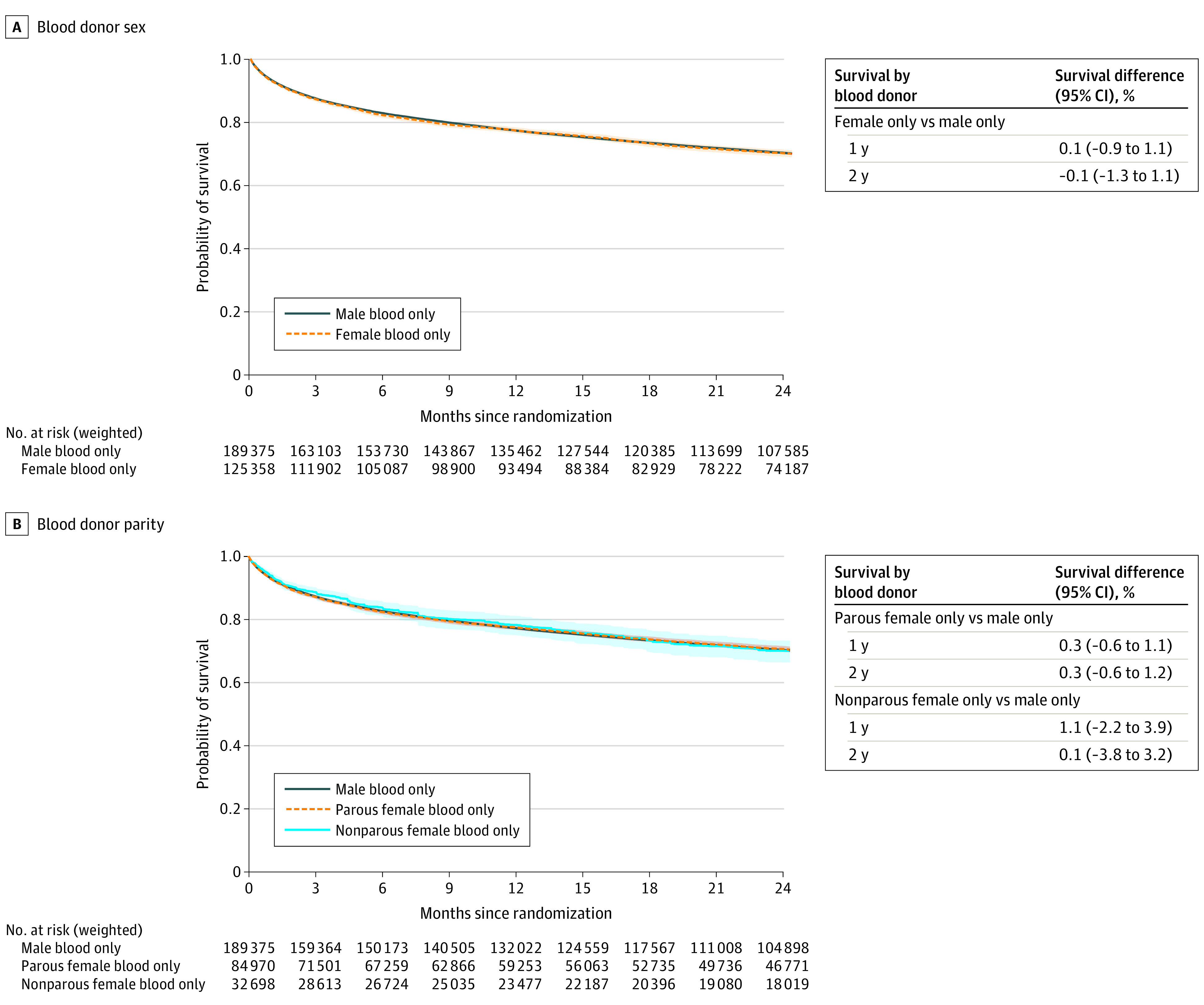
Overall Survival by Blood Donor Sex and Parity The shaded area of the graphs indicates 95% CIs.

There was no statistically significant difference in overall survival at 2 years between female and male donor strategies (survival difference, −0.1%; 95% CI, −1.3% to 1.1%; Figure 2A). Exposure assessment was truncated after the fifth red blood cell unit, which was the 99th percentile of the number of transfused units for patients receiving blood from female donors.

### Parous Female and Nonparous Female vs Male Donors

Figure 2B shows the overall survival for parous and nonparous female vs male donor strategies. Exposure assessment was truncated after the third red blood cell unit, which was the 99th percentile in the nonparous female donor arm. At 2 years, there were no statistically significant differences in overall survival comparing parous female donors (survival difference, 0.3%; 95% CI, −0.6% to 1.2%) and nonparous female donors (survival difference, 0.1%; 95% CI, −3.8% to 3.2%) with male donor strategy.

### Sex-Discordance, Subgroup, and Sensitivity Analyses

**Figure 3.**
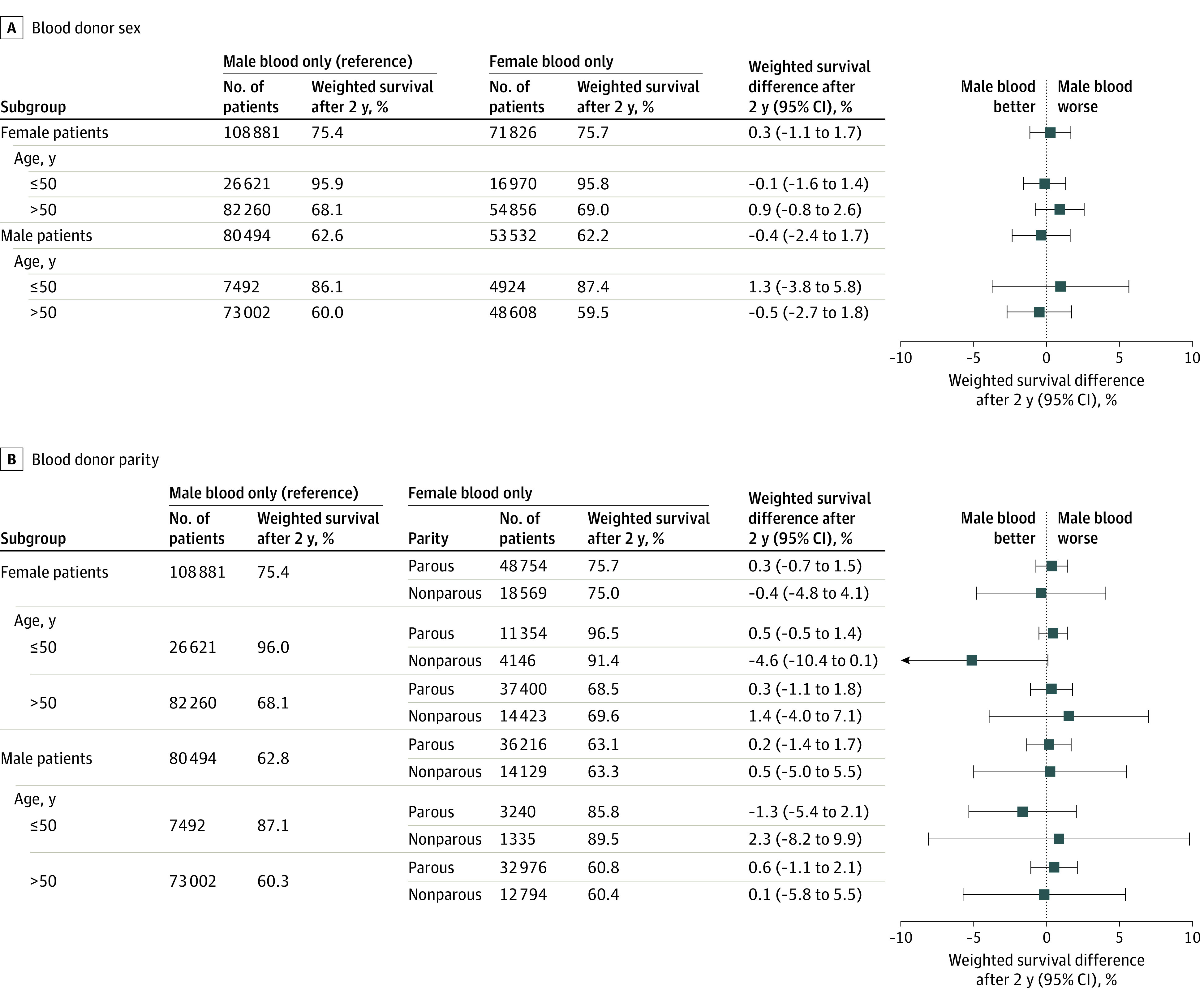
Subgroup Analyses by Patient Sex and Age

The survival difference after 2 years for the 2 sets of strategies in subgroups of patient sex and age was examined (Figure 3). There was no statistically significant difference in any subgroup. In sensitivity analyses, exposure was assessed to the 99.9th percentile, corresponding to the first 7 and 4 units for donor sex and parity, respectively. No statistically significant survival differences were found for any treatment strategy (eFigures 6 and 7 and eTable 3 in the Supplement).

## Discussion

In this nationwide, natural experiment of adult patients who received red blood cell transfusions over an 8.5-year period, patient mortality did not differ in relation to the sex or parity of the blood donor. These findings were consistent in subgroups of patient sex and age. We demonstrated theoretical and empirical support for donor parity and sex being naturally assigned as-if randomized to patients undergoing transfusion, which we exploited to emulate a randomized trial. Furthermore, we demonstrated that red blood cell transfusions from female donors incurred a higher risk for additional transfusions owing to lower donor hemoglobin counts in female donors. We applied a suitable analytical framework that mitigated bias caused by the difference in red blood cell unit efficacy and were able to estimate the effect comparable to a randomized clinical trial. Given the use of nationwide blood bank data over an 8.5-year period with exposure and outcome ascertainment from nationwide health registers, these estimates were both precise and should be widely generalizable.

Previous observational studies have been conflicting. Chassé et al^6^ reported a survival benefit among recipients of red blood cell units from male donors compared with female donors, whereas Caram-Deelder et al^8^ reported lower survival for male patients undergoing transfusion with red blood cells from ever-pregnant donors but not never-pregnant female donors. Several studies and 1 meta-analysis have shown increased mortality for sex-discordant red blood cell transfusions.^7,9,10,11^ However, these studies have been criticized for residual confounding owing to model misspecification,^6^ large proportion of missing data,^8^ and introducing selection bias through informative censoring.^36,37^ These limitations were addressed by a trinational study that reported mostly null results.^12^ Still, results from the trinational study were partly inconsistent, and one of the cohorts reported increased mortality for sex-discordant transfusions in 1 subgroup.^12^

However, previous studies did not consider that female donors have lower hemoglobin counts and that their units contain a lower dose of hemoglobin. This creates treatment-confounder feedback, a type of time-dependent confounding that cannot be accounted for using standard regression models.^15,22,31,32,33,34^ Because previous observational studies have used standard regression models, they are to some extent inevitably biased toward or away from the null, with an unpredictable net effect, and are difficult to interpret causally.^38^ The present study used cloning, censoring, and weighting, a well-recognized method within pharmacoepidemiology, to remedy bias owing to treatment-confounder feedback.^15,22,31,32,33,34,38,39^ These results are also consistent with results from a yet-published randomized trial that did not show a mortality difference in relation to donor sex.^40,41^

### Limitations

The study has limitations. We did not assess extreme cases or exposures that did not occur in a real-world setting and truncated exposure at the 99th percentile of the number of transfusions from the same donor sex or parity. However, sensitivity analyses with exposure assessment up to the 99.9th percentile also yielded null findings. We were not able to explicitly consider special cases of phenotype-matched transfusions, although this was rare and should not be contingent on donor sex or parity and is therefore unlikely to result in uncontrolled confounding. We did not evaluate other blood products and did not differentiate between red blood cell units with and without leukoreduction because the latter was uncommon. Lastly, we did not include pediatric patients or evaluate outcomes other than death.

## Conclusions

 In this nationwide cohort study involving a natural experiment, patients undergoing transfusion with blood from female or parous donors did not have a higher 2-year mortality compared with recipients of blood from male donors. Red blood cell units from female donors contained less hemoglobin compared with units from male donors, which is a hitherto unrecognized source of confounding that cannot be accounted for using standard regression models and, to our knowledge, has not been considered in previous studies.

## Notes

Supplement. **eMethods 1.** Non-technical, graphic summary of analytical method **eTable 1.** Target Trial Protocol **eTable 2.** Descriptive statistics of exposure and outcome
eMethods 2.
**eFigure 1.** Directed Acyclic Graph for our study design **eFigure 2.** Absolute standardized mean differences of baseline patient characteristics for donor sex treatment strategies **eFigure 3.** Absolute standardized mean differences of baseline patient characteristics for donor parity treatment strategies **eFigure 4.** Estimated versus observed probabilities of receiving a red-cell unit in concordance with assigned treatment strategy for donor sex **eFigure 5.** Estimated versus observed probabilities of receiving a red-cell unit in concordance with assigned treatment strategy for donor sex and parity **eFigure 6.** Kaplan-Meier Curves (99.9th percentile truncation) **eFigure 7.** Subgroup analyses (99.9th percentile truncation) **eTable 3.** Descriptive statistics of exposure and outcome (99.9th percentile truncation)
