# Supplementary material for: Mortality Among Patients Undergoing Blood Transfusion in Relation to Donor Sex and Parity: A Natural Experiment
Source: JAMA Intern Med. 2022 Jun 13;182(7):747–56. doi: 10.1001/jamainternmed.2022.2115 (PMC9194724; doi:10.1001/jamainternmed.2022.2115)
Supplement: Supplement. — eMethods 1. Non-technical, graphic summary of analytical method eTable 1. Target Trial Protocol eTable 2. Descriptive statistics of exposure and outcome eMethods 2. eFigure 1. Directed Acyclic Graph for our study design eFigure 2. Absolute standardized mean differences of baseline patient characteristics for donor sex treatment strategies eFigure 3. Absolute standardized mean differences of baseline patient characteristics for donor parity treatment strategies eFigure 4. Estimated versus observed probabilities of receiving a red-cell unit in concordance with assigned treatment strategy for donor sex eFigure 5. Estimated versus observed probabilities of receiving a red-cell unit in concordance with assigned treatment strategy for donor sex and parity eFigure 6. Kaplan-Meier Curves (99.9th percentile truncation) eFigure 7. Subgroup analyses (99.9th percentile truncation) eTable 3. Descriptive statistics of exposure and outcome (99.9th percentile truncation) [file jamainternmed-e222115-s001.pdf]

Supplemental Online Content

Zhao J, Sjölander A, Edgren G. Mortality among patients undergoing blood transfusion in relation to donor sex and parity: a natural experiment. *JAMA Intern Med*. Published online June 13, 2022. doi:10.1001/jamainternmed.2022.2115

eMethods 1. Non-technical, graphic summary of analytical method

eTable 1. Target Trial Protocol

eTable 2. Descriptive statistics of exposure and outcome

eMethods 2.

eFigure 1. Directed Acyclic Graph for our study design

eFigure 2. Absolute standardized mean differences of baseline patient characteristics for donor sex treatment strategies

eFigure 3. Absolute standardized mean differences of baseline patient characteristics for donor parity treatment strategies

eFigure 4. Estimated versus observed probabilities of receiving a red-cell unit in concordance with assigned treatment strategy for donor sex

eFigure 5. Estimated versus observed probabilities of receiving a red-cell unit in concordance with assigned treatment strategy for donor sex and parity

eFigure 6. Kaplan-Meier Curves (99.9th percentile truncation)

eFigure 7. Subgroup analyses (99.9th percentile truncation)

eTable 3. Descriptive statistics of exposure and outcome (99.9th percentile truncation)

This supplemental material has been provided by the authors to give readers additional information about their work.

© 2022 Zhao J et al. *JAMA Intern Med.*

eMethods 1. Non-technical, graphic summary of analytical method

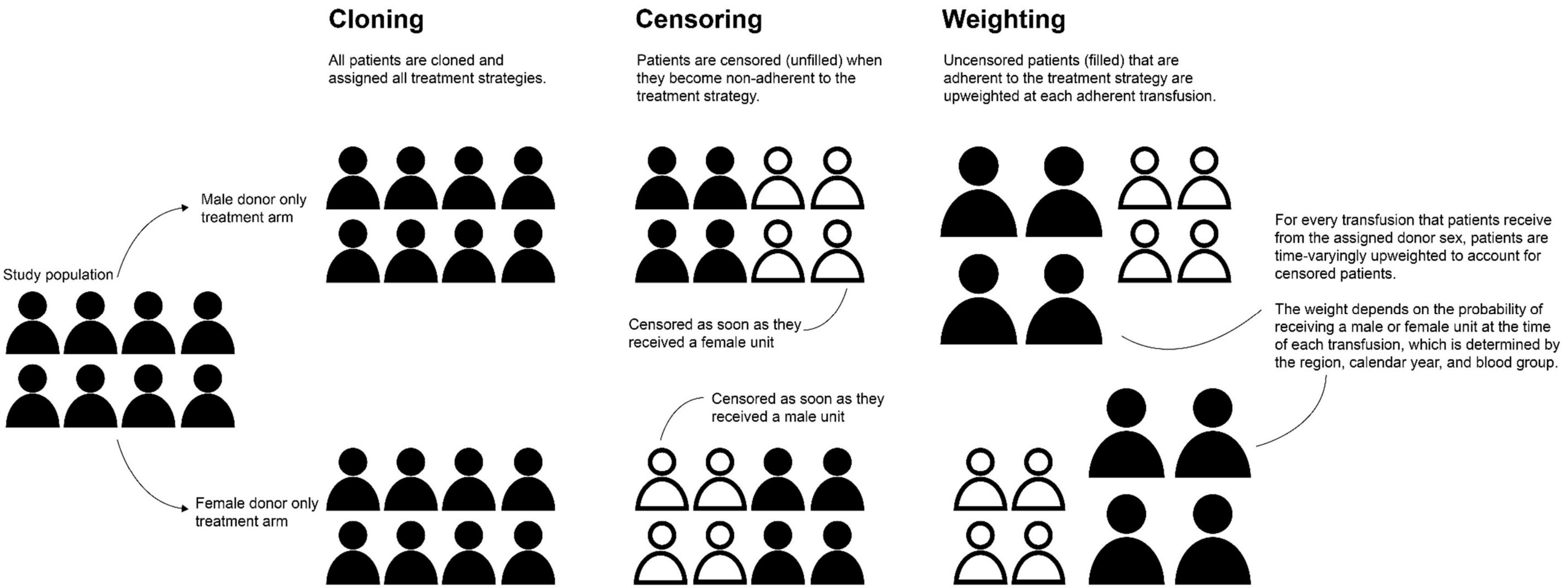

Each patient is initially cloned and assigned to all treatment arms. Each clone is subsequently censored when they become nonadherent to the treatment strategy. In practice, this results in treatment assignment being equal to the donor characteristic of the first red-cell unit. To accommodate for the censored patients, patients who continue to receive transfusions from the assigned donor characteristic are upweighted. These three steps are identical to those in a marginal structural model. The weight for non-censored patients is the inverse of the probability of being uncensored, which is simply the inverse of the probability of receiving a transfusion from the assigned donor characteristic.

© 2022 Zhao J et al. *JAMA Intern Med.*

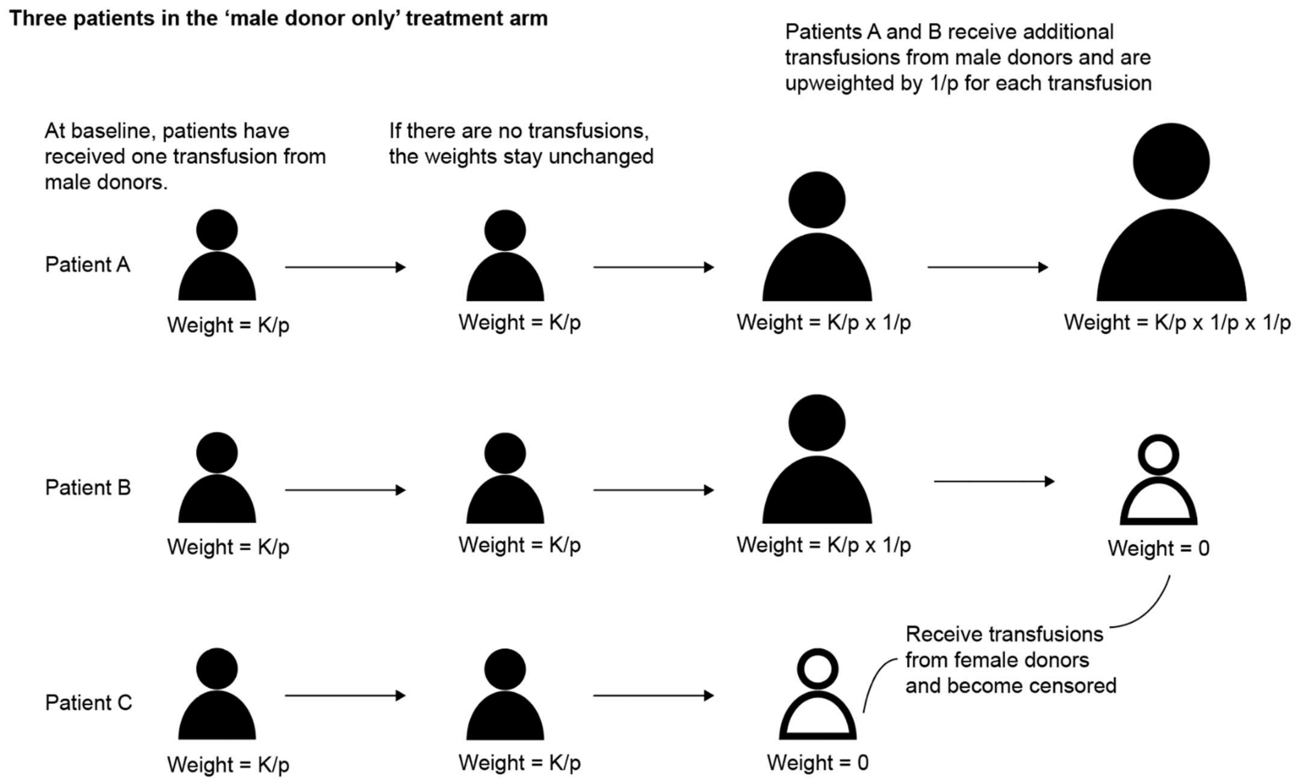

As an example, three patients (A, B, C) have been assigned to the male donor only treatment arm. At baseline, each patient has received a transfusion from a male donor and has the weight of a constant K (see eMethods 2 section 4) multiplied with the inverse of the probability of receiving a male unit (p), i.e., K/p. At each transfusion, patients become upweighted with a factor $1/p$. The weight is constant between transfusions. When patients B and C later receive a unit from a female donor, they are censored and receive a weight of 0. Inverse probability weighting mitigates bias due to treatment-confounder feedback between donor sex and the cumulative number of transfusions, because it balances the cohort at each time point instead of across all of follow-up at once. Traditional methods such as Cox regression are susceptible to bias due to treatment-confounder feedback even in the setting of random exposure allocation.

© 2022 Zhao J et al. *JAMA Intern Med.*

eTable 1. Target Trial Protocol

	Target trial	Emulated trial
**Eligibility criteria**	Adult patients between 18-90 years who receive red-cell transfusions between years 2010 and 2017, with no prior history of red-cell transfusions.	Same, with transfusion history assessed using nationwide registers with coverage up to 50 years prior to baseline.
**Exclusion criteria**	Infeasible scenarios such as massive transfusion protocols, due to differential feasibility (i.e., greater difficulty finding enough blood with less common donor characteristics)	Most patients with massive transfusion protocols will be naturally censored due to non-adherence in an observational dataset. However, to avoid unbalance of extreme cases between treatment groups due to differential probabilities of receiving units from blood donors with a certain characteristic (i.e., it will be more common to have outliers/extreme cases in the group with the more common donor characteristic), we truncated the number of units at the 99th percentile of the least common group, and at the 99.9th percentile in sensitivity analysis.
**Treatment strategies**	For donor sex:<br>1. Blood from male donors only<br>2. Blood from female donors only<br><br>For donor parity:<br>1. Blood from male donors only<br>2. Blood from non-parous female donors only<br>3. Blood from parous female donors only	Same
**Assignment procedures**	Randomly assigned at baseline.	All patients will be cloned and assigned all treatment strategies. In practice, blood donor characteristic of first red-cell transfusion is the assigned treatment strategy, which was assigned as-if randomized.
**Follow-up period**	Starts at randomization and ends at death, emigration, transfusion from unidentified donor, June 30, 2018, or 2 years after baseline, whichever occurs first.	Same
**Outcome**	Death within 2 years of baseline	Same
**Causal contrasts of interest**	The effect of initiating and adhering to a treatment strategy.	Same
**Analysis plan**	Inverse probability weighting to adjust for censoring for non-adherence.	Same

© 2022 Zhao J et al. *JAMA Intern Med.*

eTable 2. Descriptive statistics of exposure and outcome

	Donor sex		Donor sex/parity		
**Characteristic**	**Male donor only (N=189 375)**	**Female donor only (N=125 358)**	**Male donor only (N=189 375)**	**Nonparous female donor only (N=32 698)**	**Parous female donor only (N=84 970)**
Number of red-cell transfusions					
Compliant transfusions — no.	411 153	211 138	512 338	407 94	138 590
Weighted compliant — median (IQR)	2 (2-4)	3 (2-5)	2 (2-4)	2 (1-3)	2 (1-3)
Unweighted compliant 99th percentile — no.	6	5	6	3	4
Events — no. (%)					
Compliant deaths	23 434 (12)	10 411 (8)	29 460 (16)	1666 (5)	6727 (8)
Compliant censoring	12 390 (7)	6448 (5)	13 996 (7)	967 (3)	4093 (5)
Noncompliant	88 048 (46)	72 134 (58)	72 966 (39)	21 886 (67)	50 296 (59)

Weighted transfusions were calculated with the last non-zero weight for each person.

Numbers for male donor strategy differ because donor sex was assessed for first 5 red-cell units and donor parity was assessed for first 3 units.

© 2022 Zhao J et al. *JAMA Intern Med.*

eMethods 2

Below, we motivate the analytical approach for donor sex, but it is readily generalizable to donor parity. Previous studies of the effects of donor sex and parity on patient outcomes have mostly used variations of time-dependent Cox proportional hazards models. As explained below, such models are unsuitable in this scenario due to treatment-confounder feedback and may result in biases in unpredictable directions.

In Section 1, we formalize our study and assumptions using a Directed Acyclic Graph. In Section 2, we demonstrate how previous studies may be biased due to treatment-confounder feedback. Further, we empirically demonstrate that treatment-confounder feedback is present in longitudinal studies of blood donation where donor sex is or affects the treatment group, caused by lower hemoglobin count in female donors. In Section 3, we empirically evaluate our assumptions in Section 1 by comparing patient characteristics across treatment groups, with and without inverse probability of treatment weighting to accommodate for center-effects. In Section 4, we argue that donor sex is binomially distributed given assumptions from 1 and demonstrate our final inverse probability weighted analysis. In Section 5, we empirically verify the binomial distribution from Section 4.

1. Formalization of the study design and assumptions

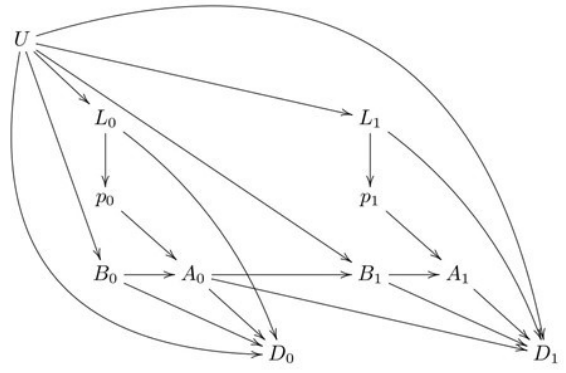

eFigure 1. Directed Acyclic Graph for our study design

Let B_t and p_t be the number of received blood units and the probability of receiving a male blood unit at time t, respectively. Let A_t be our treatment variable, which we define as the total number of received blood units from male donors at time t. Note that, given B_t, the total number of received blood units from female donors is $B_t - A_t$. In multicenter randomized controlled trials that span large time periods or adaptive randomized trials, the randomization probabilities and patient population may vary across centers and time. Analogously, in our non-randomized observational study, the probability of receiving a male blood unit may vary across patients' blood group, geographical regions and calendar year. Let L_t be the vector that includes these measured "center-specific" characteristics. Finally, let D_t be the indicator of the outcome (death) occurring at time t. The Directed Acyclic Graph (DAG) in eFigure 1 illustrates the relation between these variables for two time points $t = 0$ and $t = 1$, with obvious generalization to several time points. The variable U represents all (unmeasured) factors that may have a direct effect on center characteristics (L_t), the number of units received (B_t) and death (D_t), such as unmeasured comorbidity, patient frailty, and geodemographic differences. In practice, some of these factors may be time-varying as well, but this does not affect the following rationale and for brevity we have depicted U as time-constant in Figure 1. Note that the DAG does not include direct effects of (L_0, p_0, B_0) on (L_1, p_1, B_1), or direct effects of A_0 on (L_1, p_1). These simplifications are just for brevity; none of our arguments or analyses below rely on these effects being absent.

Note that the DAG allows for a direct effect for the sex of the blood donor in the previous time period on the number of transfused units in the next time period, i.e., an arrow from A_0 to B_1,. We argue in Section 2 below that such an effect is present, and that the consequence of this effect is that the standard regression analyses used in previous studies are likely biased.

Importantly, the DAG assumes that the probability of receiving a male blood unit (p_t) is determined solely by center characteristics (L_t), i.e., blood group, geographical region, and calendar year. This assumption follows by the way blood units are stored and distributed to patients. Since blood donor sex is not recorded on blood units and is not considered when allocating units to specific patients, it should not be possible for the probability of receiving a unit with a specific donor sex to be associated with patient characteristics. In other words, there are

no other common causes of death and the probability of receiving red-cells from male donors other than through blood group, geographical region, and calendar year (L_t). For clarity, we have explicitly used the notation p_t is explicitly identified in the DAG in order to continue using the same notation in subsequent expressions.

From this reasoning, it follows that the measured variables number of transfusions (B_t) and blood group, geographical region, and calendar year (L_t) are sufficient to completely block all confounding ("back-door") paths between transfusions from a specific donor sex (A_t) and death (D_t). This assumption of no unmeasured confounding for the treatment and the outcome is crucial for our inverse probability weighted analysis to provide unbiased estimates of the causal effect of blood donor sex on mortality. In Section 3 we argue that, although this assumption is not logically guaranteed, it is indeed highly plausible under our study design. Furthermore, we evaluate this assumption empirically in Sections 3 and 5.

2. Treatment-confounder feedback due to donor hemoglobin count, and its consequences for previous studies

To see how the direct effect of donor sex on the number of units transfused (i.e. A_0 on B_1) may lead to bias in standard analyses, suppose that we consider the joint effect of A_0 and A_1 on D_1. We would then need to adjust for B_1, since this variable acts as a confounder for A_1 and D_1. However, standard adjustments for B_1, blocks the path $A_0 \rightarrow B_1 \rightarrow D_1$, thus removing some of the mediated effect of A_0 on D_1. Worse, standard adjustment for B_1 opens the backdoor path $A_0 \rightarrow B_1 \leftarrow U \rightarrow D_1$ at which B_1 acts as a collider, thus inducing a statistical association between A_0 and D_1, even in the absence of a causal effect. Because standard adjustment methods both blocks a part of the effect of interest and opens up a backdoor path, the net statistical association is unpredictable and can act either towards or away from the null, depending on the balance of the two effects.

With some variations, previous studies have compared patients who up until a given time have received the same cumulative number of transfusions, e.g. patients that have received 3 units from male donors compared to 3 units from female donors. In other words, previous studies have adjusted for B_t as the time-dependent cumulative number of transfusions, which, under the presence of treatment-confounder feedback, may create bias both towards and away from the null with an unpredictable net statistical effect.

In our study, treatment-confounder feedback is likely present due to differences in donor hemoglobin. As low patient hemoglobin count is the major driver for transfusion need, and since female donors have lower hemoglobin count on average, patients receiving female units only at some point in time will need more units at subsequent time points. To assess this empirically, we calculated the relative risk of receiving an additional transfusion within 24 hours depending on the donor sex of the previous transfusions. Because double-unit transfusions were commonplace in Sweden during the study period, we assessed the donor sex of the first two transfusions. We restricted the analysis to patients that received two red-cell units within the first 6 hours of the first transfusion (62% of cohort). We then used a log-binomial regression model to estimate the relative risk of receiving another transfusion within 18 hours of the second transfusion, adjusting for center characteristics as the interaction of blood group, region, their main effects, and calendar year. To show the role of donor hemoglobin, we additionally adjusted for the mean donor hemoglobin of the two units. All numerical values (donor hemoglobin, calendar year, patient age) were modelled as restricted cubic splines with three knots, using knot placement as suggested by Harrell at the 10th, 50th, and 90th percentile. To additionally assess the relative risk of receiving transfusions based on donor hemoglobin counts only, we compared patients that received transfusions from donors who had hemoglobin counts in the 10th percentile compared to the 90th percentile. Death was not considered a competing event because it was rare (<1%).

Using the method above, we found that the median hemoglobin count for female donors was 13.5 g/dl (interquartile range [IQR], 13.0 to 14.0 g/dl) and for male donors 14.9 g/dl (IQR, 14.4 to 15.5 g/dl). For all donors, the 10th percentile for donor hemoglobin count was 13.3 g/dl and the 90th percentile was 15.4 g/dl. Receiving red-cell units from female donors only was associated with a 12% increased risk of an additional transfusion within 24 hours, as compared to male donors only (relative risk, 1.12; 95% CI, 1.08 to 1.17). Further, receiving red-cell units from donors with lower hemoglobin count was associated with 20% increased risk (95% CI, 1.15 to 1.26). However, the difference between female and male donors was no longer significant after adjusting for donor hemoglobin counts (relative risk, 1.03; 95% CI, 0.98 to 1.08). Additionally adjusting for patient sex or age did not affect estimates.

In other words, the increased risk of receiving additional transfusions among patients who received exclusively female units is driven by differences in donor hemoglobin count between male and female donors. Translating this to our notation above, we find that a low value of A_0 (in relation to B_0) may induce a higher value of B_1; this both shows the mechanism for and the presence of treatment-confounder feedback. It is therefore inappropriate to

© 2022 Zhao J et al. *JAMA Intern Med.*

adjust for the cumulative number of transfusions as in previous studies, because we expect patients that received female units to need more units.

3. Assumption of no unmeasured confounding

The crucial assumption in Figure 1 is that there are no unmeasured confounders for the treatment and the outcome, as there are no direct arrows from U to p_t (or from U to A_t). Under this assumption, causal treatment effects can be estimated, even if standard estimation methods are generally biased due to treatment-confounder feedback.

We motivate this assumption based on the mechanism for how blood units are handled and distributed, combined with empirical data. Firstly, we know that red-cell units are selected for a given patient based on availability in a given place at a given time, as well as on blood group of the donor in question, but otherwise obey a first-in-first-out policy. Since neither donor sex nor donor parity is recorded on the blood units, it should not be possible to sort or select units based on these factors.

Secondly, we show empirically in Table 1 and 2 that pre-transfusion patient characteristics stratified on donor sex or parity are distinctly similar. However, based on *a priori* knowledge, there could be "center-effects" even if we do not observe large differences in Tables 1 and 2, due to patient's blood group, geographical region, and calendar year. For example, it is possible that some regions may have a larger supply of male blood, either because these regions have been particularly successful in recruiting male donors or simply because the proportion of males is higher than the proportion of females in these regions. If the survival of recipients also varies across regions (e.g., because average age may be higher in non-urban areas and the most advanced clinical procedures may be centralized to university hospital clinics in select regions), then the region where the recipient was treated may act as a confounder in our study. With similar arguments, blood group and calendar year may also act as confounders.

To control for such center-effects, we estimated center-specific probabilities of receiving red-cells from male donors, i.e., probabilities adjusted for the center-specific characteristics L_t. These were estimated non-parametrically using administrative data of all transfusions nationwide, stratified by patients' blood group, region, and calendar year. For clarity, this is analogous to regression adjustment for calendar year, geographical region, and blood group. We then proceeded to construct inverse probability of treatment weights at baseline, given by

$$W_{treat,i} = \frac{1}{p(A_i|L_i)},$$

where A_i is donor sex of the received unit at baseline and L_i are the center-specific characteristics corresponding to this donation. To evaluate the balance of patient characteristics before and after weighting, we assessed the absolute standard mean differences for key patient characteristics at baseline. Absolute standard mean differences were calculated as the absolute difference of the mean of each characteristic between persons assigned male donor only compared to female or parous/nonparous females divided by the variance of the entire population. Comparing male donors only to female donors only treatment arms, unweighted differences were smaller than 5% for calendar year, patient age, and most blood groups and regions, which decreased after weighting to less than 2% at most (eFigure 2). For prevalence of comorbidities, including total Elixhauser comorbidity index score had at most an unweighted difference of 1.7%, which decreased to at most 0.7% after weighting. Similar results were found for comparing donor parity (eFigure 3).

Taken together, we do not to observe meaningful differences across treatment arms at baseline in terms of patient demographics, transfusion indication, geographical distribution, or any of the 31 categories of comorbidities, especially after considering center-specific effects by weighting with center-specific inverse probabilities of treatment. Also considering the known allocation mechanism which only considers blood group, and that donor sex and parity is not known by the clinicians administering the transfusions and is not considered by the blood banks allocating the units, it is hard to imagine that there exist other unmeasured factors with substantial influence on both the distribution of male and female donor blood in the supply and the survival of recipients. We interpret this as strong support – both empirical and theoretical – for the assumption of donor sex and parity being as-if randomized considering region, calendar year, and recipient blood group. Sensitivity analyses where we additional considered the patients' age, sex, and transfusion indication did not affect estimates for the proportion of male/female donors (see Section 5).

© 2022 Zhao J et al. *JAMA Intern Med.*

eFigure 2. Absolute standardized mean differences of baseline patient characteristics for donor sex treatment strategies

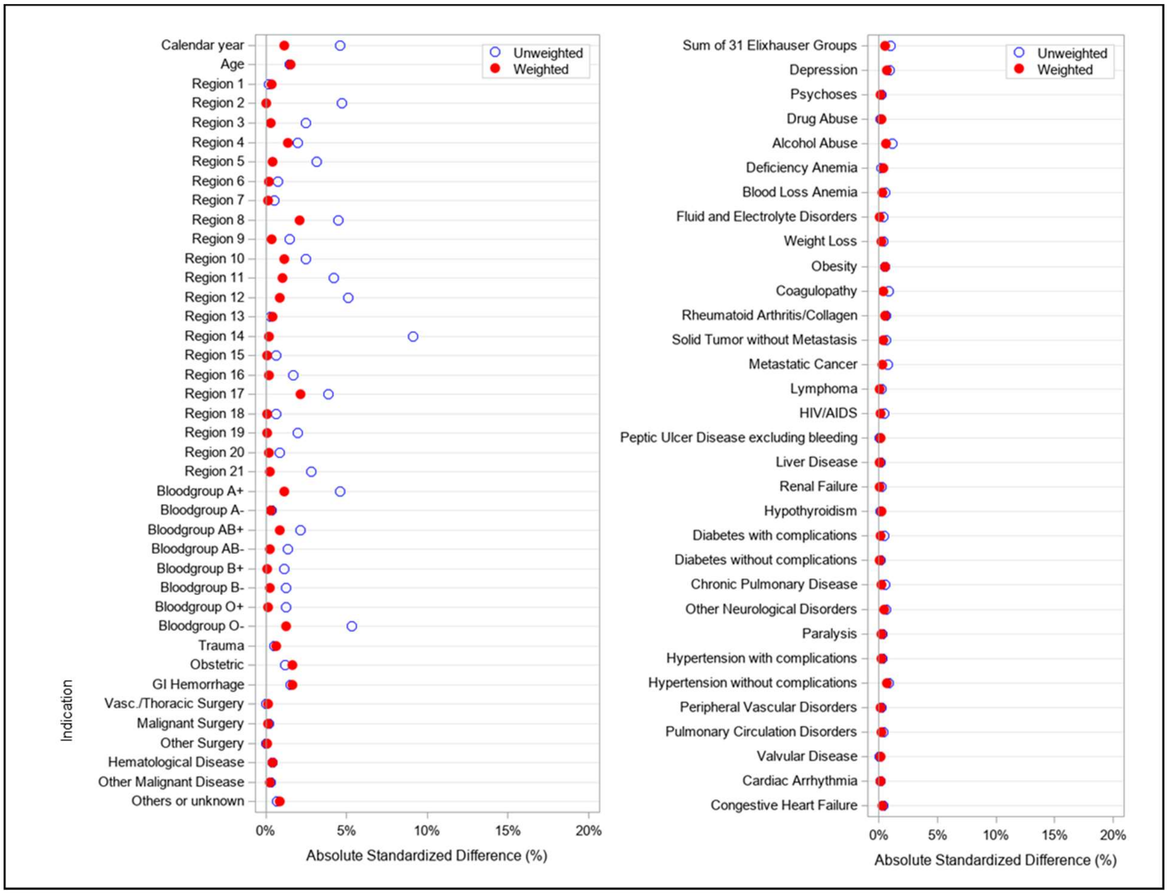

© 2022 Zhao J et al. *JAMA Intern Med.*

eFigure 3. Absolute standardized mean differences of baseline patient characteristics for donor parity treatment strategies

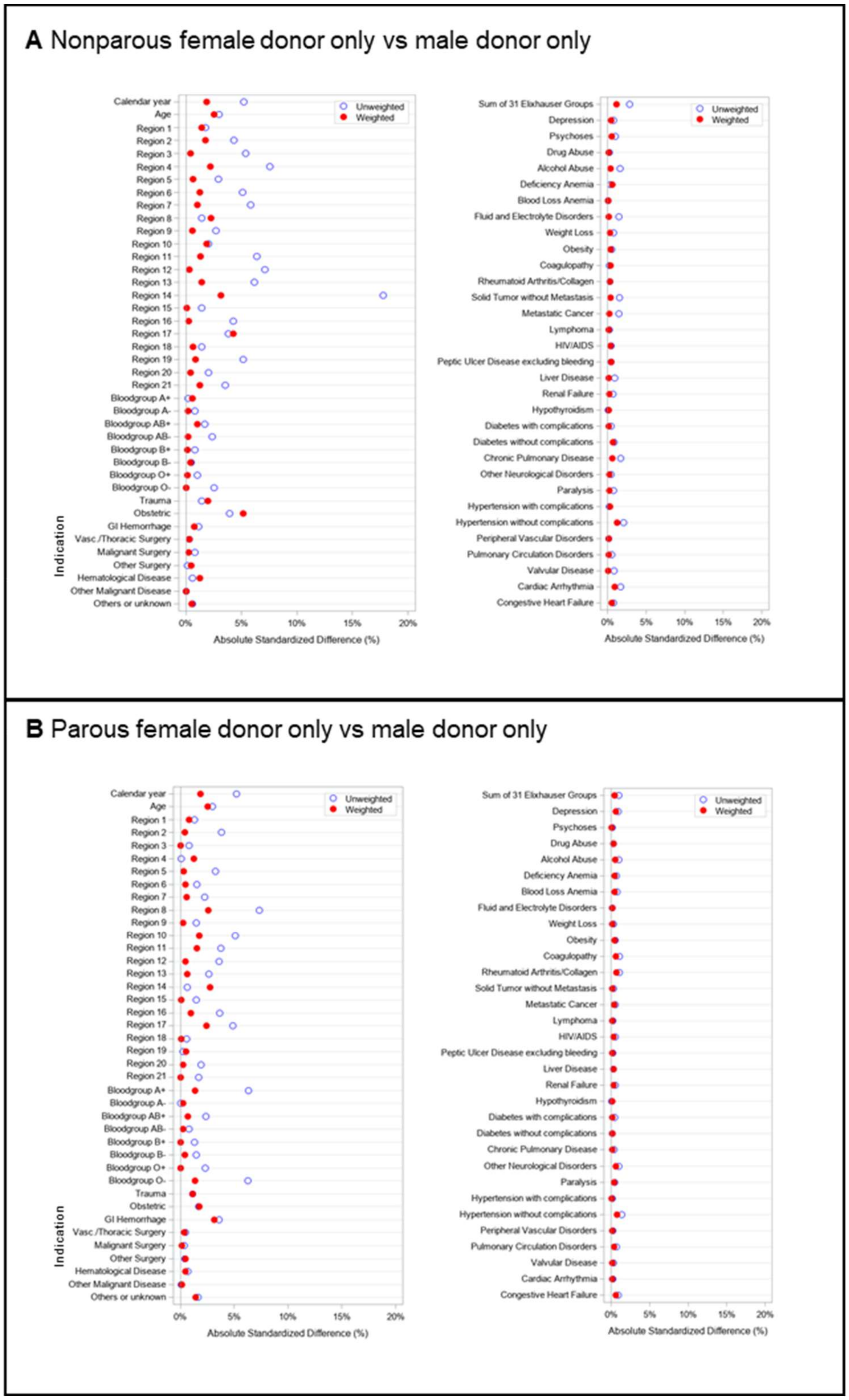

© 2022 Zhao J et al. *JAMA Intern Med.*

4 Inverse probability weighted analysis

To mitigate the issues of treatment-confounder feedback, we used an inverse probability weighted analysis for dynamic treatment strategies, as proposed by Hernan et al. (Basic Clin Pharmacol Toxicol, 2006). Conceptually, we created two pseudo-populations where in one group everyone receives only male units and in one group everyone only receives female units, as in a randomized controlled trial. This is achieved by artificially censoring patients at the point of non-adherence and upweighting adherent patients by the inverse probability of receiving a transfusion in line with the assigned treatment.

For this weighted analysis, follow-up time has to be discretized into time intervals. To capture the iterative decision-making process that is the source of the treatment-confounder feedback, analyses must be conducted in clinically relevant time intervals. Because weights are constant during a time interval, we assume that everything during each time interval occurs simultaneously. It will therefore not be possible to disentangle the cause and effect of multiple decision-making processes during longer time-intervals such as days or weeks. Based on clinical experience of the time needed to order and administer a transfusion, and to evaluate its effect, we conducted our analyses in intervals of one hour. The timing of transfusions was based on the recorded time of transfusion from local blood bank databases.

As argued above, A_t only depends on B_t and p_t. Specifically, given B_t and p_t, A_t has a binomial distribution

$$p(A_t = a_t | B_t, p_t) = \binom{B_t}{a_t} {p_t}^{a_t} (1 - p_t)^{B_t - a_t}$$

For each time interval t and recipient i, we measured the number of received blood units $B_{t,i}$ and the treatment $A_{t,i}$. We next estimated the probability $p_{t,i}$ non-parametrically for each time interval t and patient i, as a function of region, calendar year, and patient's blood group. In other words, we control for L_t through $p_{t,i}$. We used these to compute inverse-probability-of-censoring (IPC) weights for each time interval t and recipient i, given by

$$IPCW_{t,i} = \frac{K}{\prod_{k=0}^{t} p\left(A_{k,i} | B_{k,i}, p_{k,i}\right)}$$

Here, K is the baseline scaling factor to restore the sum of baseline weights back to the number of physical persons.

Finally, to estimate survival we computed a weighted Kaplan-Meier curve for each treatment strategy under consideration, using the IPC weights obtained from the previous step. For these curves, we censored recipients whenever they no longer adhered to the treatment specified by the strategy. For instance, for the Kaplan-Meier curve corresponding to the strategy "blood from male donors only" we censored recipients at the first time t when they received a blood unit from a female donor (i.e., when $A_{t,f} > 0$). In the absence of unmeasured confounding for the treatment and the outcome, as in Figure 1, then the obtained Kaplan-Meier curves have causal interpretations. Specifically, they unbiasedly estimate the survival functions that would be observed in a large trial with full compliance, where patients are randomly assigned to either treatment strategy at baseline. Bootstrapping with 1,000 runs was used to calculate confidence intervals that also consider the uncertainty of the weights.

5 Evaluation of binomial distribution, independence, and sensitivity analyses

As derived in 3.4, our analysis relies on an assumed binomial distribution for donor sex, dependent on only the number of transfusions and the center-specific probability of receiving a male donor unit. We assessed this assumption empirically by calculating the expected probability of receiving the assigned unit type based on a binomial distribution per time interval and compared it to the observed probability. In other words, we let the expected probability be the probability for the cohort raised to the power of the number of transfusions per time interval.

As a sensitivity analyses, we estimated the expected probability using a logistic regression model further incorporating patient sex, patient age (as a restricted cubic spline to account for potential non-linearity, with knots at 20, 45, 60, 75, 85), and transfusion indication as categorical variables based on a hierarchical classification using discharge ICD-codes (Auvinen et al, Transfusion 2020). Blood group and region interaction and their main effects, as well as calendar year (as a restricted spline with knots at 2011, 2013, 2015, 2017) were also included. As a confounder would need to affect both the probability of receiving a male or female unit and patient death, this directly assessed patient age, sex, and transfusion indication as confounders.

© 2022 Zhao J et al. *JAMA Intern Med.*

The estimated and observed probabilities were close and well within 95% confidence intervals. Additional covariates such as patient age, sex and transfusion indication did not affect the sex of the blood donor and estimates between non-parametric and logistic regression models were consistent up to at least two decimal units (eFigure 4). We furthermore confirmed the fit with donor parity (eFigure 5).

Taken together, we find strong empirical support for the binomial distribution arrived at in Section 4 and the underlying assumptions presented in Section 1. In other words, there are strong reasons to believe that the number of received blood units at time *t*, region, calendar year and patient blood group are sufficient for confounding control.

eFigure 4. Estimated versus observed probabilities of receiving a red-cell unit in concordance with assigned treatment strategy for donor sex

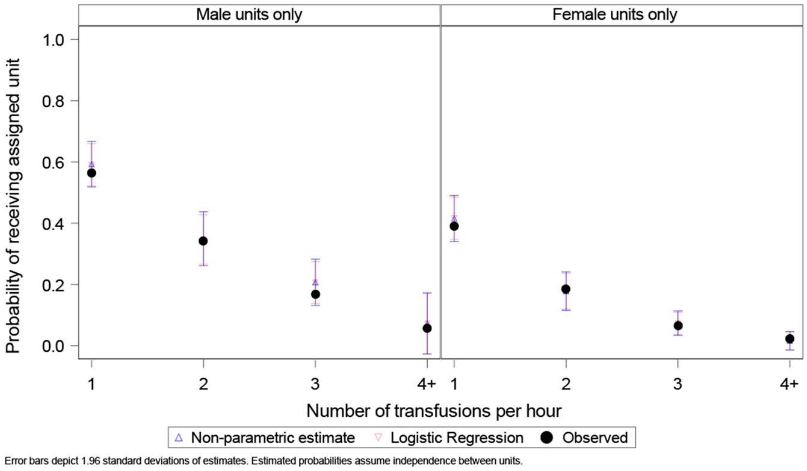

eFigure 5. Estimated versus observed probabilities of receiving a red-cell unit in concordance with assigned treatment strategy for donor sex and parity

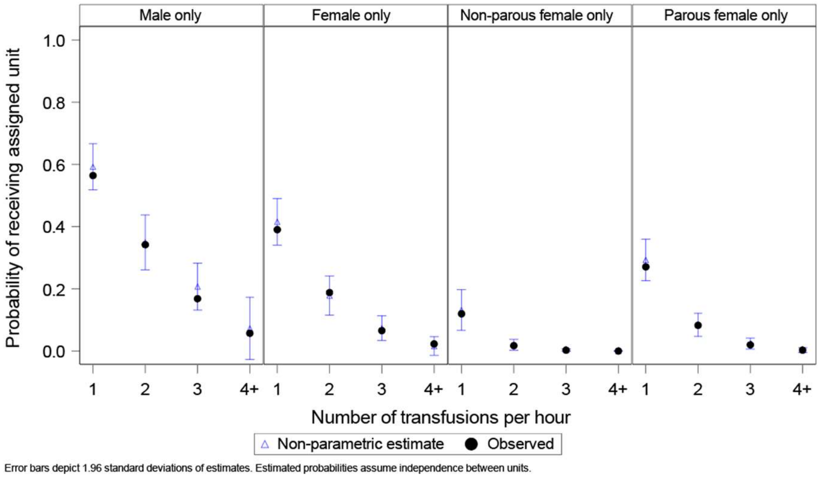

© 2022 Zhao J et al. *JAMA Intern Med.*

eFigure 6. Kaplan-Meier Curves (99.9th percentile truncation)

A Blood donor sex

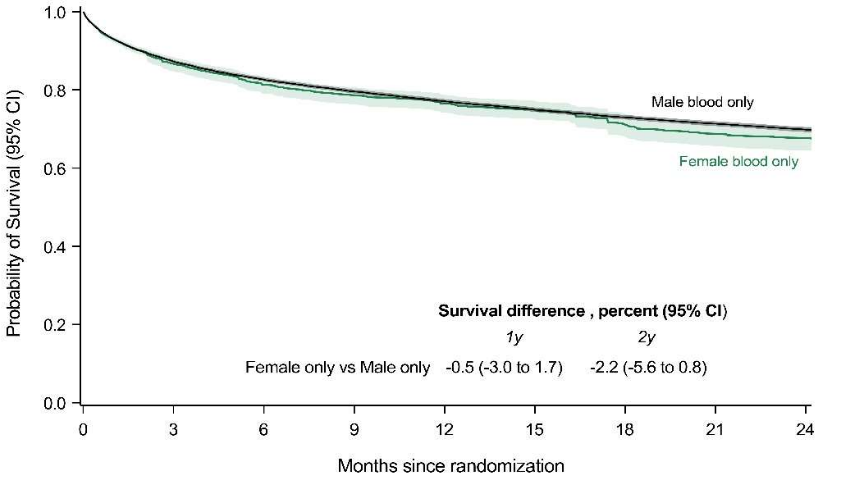

No. At Risk (weighted)									
Male blood only	189,375	165,831	156,479	147,076	138,456	130,381	123,322	116,685	110,467
Female blood only	125,358	114,108	108,198	102,305	97,686	93,363	85,445	80,452	76,439

No. At Risk (non-weighted)									
Male blood only	189,375	86,389	79,855	74,033	69,128	64,846	60,893	57,237	53,872
Female blood only	125,358	42,409	39,116	36,160	33,632	31,487	29,426	27,566	25,848

B Blood donor parity

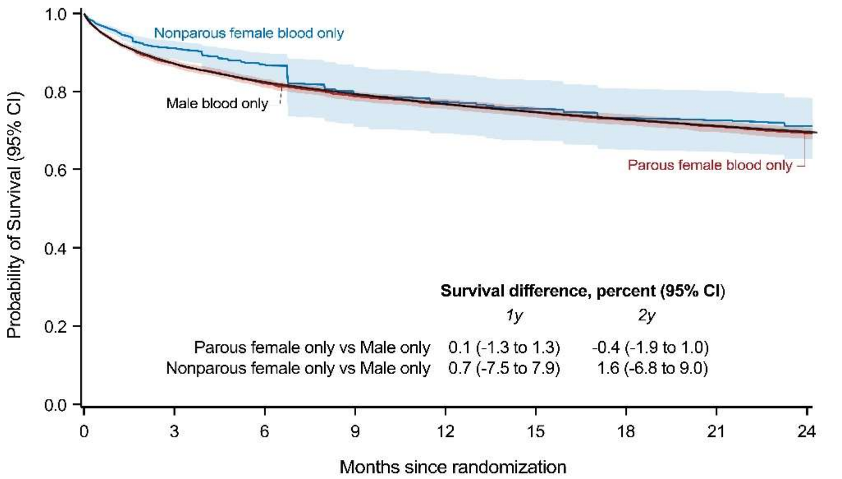

No. At Risk (weighted)									
Male blood only	189,375	164,042	154,739	144,756	136,118	128,401	121,281	114,539	108,396
Parous female blood only	84,970	77,792	73,561	69,039	65,567	62,177	58,579	55,408	51,778
Nonparous female blood only	32,698	30,107	28,445	25,566	22,960	21,802	20,435	19,226	18,123

No. At Risk (non-weighted)									
Male blood only	189,375	90,989	84,709	78,752	73,671	69,262	65,131	61.309	57.793
Parous female blood only	84,970	23,824	22,016	20,344	18,878	17,649	16,459	15,375	14,395
Nonparous female blood only	32,698	6368	5878	5444	5064	4744	4430	4135	3845

© 2022 Zhao J et al. *JAMA Intern Med.*

eFigure 7. Subgroup analyses (99.9th percentile truncation)

A Blood donor sex

Subgroup	Male blood only (ref): Number of patients	Male blood only (ref): Weighted survival after 2 years, percent	Female blood only: Number of patients	Female blood only: Weighted survival after 2 years, percent	Weighted survival difference after 2 years, percent (95% CI)
Female patients	108,881	75.0	71,826	74.9	-0.1 (-3.2 to 2.8)
≤50y	26,621	96.0	16,970	96.6	0.6 (-1.4 to 2.4)
>50y	82,260	67.5	54,856	67.8	0.2 (-3.6 to 3.8)
Male patients	80,494	62.5	53,532	57.6	-5.0 (-10.6 to 0.4)
≤50y	7,492	86.3	4,924	88.1	1.8 (-8.9 to 9.6)
>50y	73,002	59.9	48,608	54.4	-5.4 (-11.3 to 0.5)

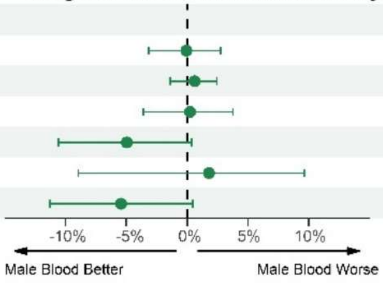

B Blood donor parity

Subgroup	Male blood only (ref): Number of patients	Male blood only (ref): Weighted survival after 2 years, percent	Female blood only by parity	Number of patients	Weighted survival after 2 years, percent	Weighted survival difference after 2 years, percent (95% CI)
Female patients	108,881	74.8	Parous	48,754	74.3	-0.5 (-2.2 to 1.4)
			Nonparous	18,569	79.7	4.9 (-1.0 to 9.8)
≤50y	26,621	95.7	Parous	11,354	95.1	-0.6 (-2.7 to 1.3)
			Nonparous	4,146	97.8	2.1 (-0.9 to 3.7)
>50y	82,260	67.5	Parous	37,400	67.3	-0.2 (-2.4 to 2.2)
			Nonparous	14,423	72.9	5.5 (-1.8 to 12.1)
Male patients	80,494	62.3	Parous	36,216	62.1	-0.2 (-2.6 to 2.3)
			Nonparous	14,129	61.8	-0.4 (-14.0 to 12.1)
≤50y	7,492	86.4	Parous	3,240	84.1	-2.3 (-9.3 to 4.2)
			Nonparous	1,335	91.9	5.5 (-5.0 to 12)
>50y	73,002	59.7	Parous	32,976	59.7	0.0 (-2.5 to 2.7)
			Nonparous	12,794	59.8	0.1 (-14.2 to 13.5)

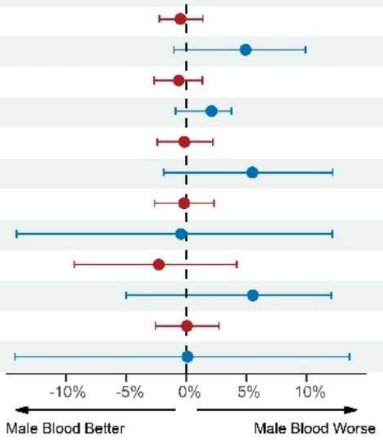

© 2022 Zhao J et al. *JAMA Intern Med.*

eTable 3. Descriptive statistics of exposure and outcome (99.9th percentile truncation)

	Donor sex		Donor parity		
**Characteristic**	**Male donor only (N=189 375)**	**Female donor only (N=125 358)**	**Male donor only (N=189 375)**	**Nonparous female donor only (N=32 698)**	**Parous female donor only (N=84 970)**
Number of red-cell transfusions					
Compliant transfusions — no.	384 735	203 915	452 292	38 842	125 927
Weighted compliant — median (IQR)	2 (2-4)	3 (2-5)	2 (2-4)	2 (1-3)	2 (2-3)
Unweighted compliant 99.9th percentile — no.	10	7	10	4	6
Events — no. (%)					
Compliant deaths	21 998 (12)	10 034 (8)	25 701 (14)	1567 (5)	5900 (7)
Compliant censoring	12 103 (6)	6369 (5)	12 847 (7)	938 (3)	3792 (4)
Noncompliant	91 205 (48)	73 040 (58)	82 867 (44)	22 144 (68)	52 511 (62)

Weighted transfusions were calculated with last non-zero weight for each person.

Numbers for male donor strategy differ because donor sex was assessed for first 7 red-cell units and donor parity was assessed for first 4 units.

© 2022 Zhao J et al. *JAMA Intern Med.*
